# Ultrasound-assisted extraction of bioactive birch (*Betula* sp.) bark triterpenoids using hydrophobic natural deep eutectic solvents

**DOI:** 10.1007/s00216-025-06174-7

**Published:** 2025-11-27

**Authors:** I. Luque-Jurado, S. Rivas, R. Lebrón-Aguilar, J. E. Quintanilla-López, M. L. Sanz, A. C. Soria

**Affiliations:** 1 Instituto de Química Orgánica General (CSIC), Juan de la Cierva, 3 28006, Madrid, Spain; 2 Instituto de Química Física Blas Cabrera (CSIC), Serrano, 119 28006, Madrid, Spain

**Keywords:** Birch (*Betula* sp.) bark, Bioactive triterpenoids, Ultrasound-assisted extraction (UAE), COSMO-RS, Hydrophobic natural deep eutectic solvents (h-NADESs), AGREEprep

## Abstract

The simultaneous extraction of high-value bioactive triterpenoids, betulin (Bet) and betulinic acid (BAc), from birch (*Betula* sp.) bark has been optimized and analytically characterized to provide an improved approach in terms of efficacy and sustainability. First, *in silico *screening of numerous conventional organic solvents, biosolvents and hydrophobic natural deep eutectic solvents (h-NADESs) via the COSMO-RS theory revealed thymol:1-octanol (molar ratio of 4:1) as the optimal extractant (Bet + BAc solvent capacity, ${C}_{\mathrm{Bet}+\mathrm{BAc}}^{\infty }$ = 21.7). Because ultrasound-assisted extraction (UAE) was found to outperform conventional solid-liquid extraction, a central composite experimental design was used to determine the UAE operating conditions that maximized the extraction yield of both target bioactives (61 °C for 24 min and 1 extraction cycle). Precise (RSD < 2.3%) and efficient extraction of Bet and BAc (recovery > 80% and 96%, respectively) was achieved by this new h-NADES UAE method, whose greenness was confirmed by an AGREEprep score of 0.76. A wide variability in the concentration of lupane-type triterpenoids (Bet: 12.09–32.17 mg g^−1^; BAc: 0.69–1.43 mg g^−1^), which is not fully dependent on the birch species considered, was observed in its application to different *Betula* sp. samples. The combination of UAE with h-NADES is a new and potential green approach for the enhanced extraction of bioactive birch bark triterpenoids for application in different fields.

**Graphical Abstract:**

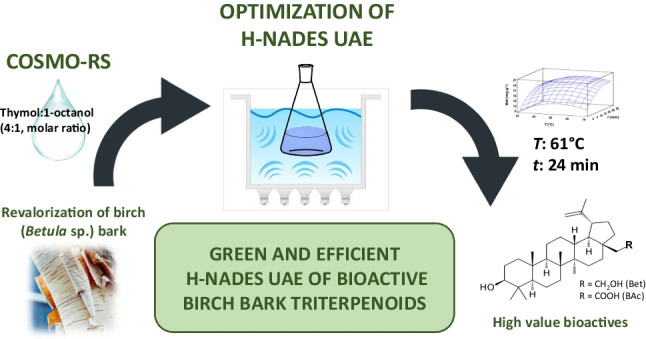

**Supplementary Information:**

The online version contains supplementary material available at 10.1007/s00216-025-06174-7.

## Introduction

The efficient and sustainable extraction of high-value bioactive compounds from biomass is not only a potential strategy for the revalorization of forestry byproducts [1–6] but also a mandatory sample preparation step prior to analysis.

Among the wide variety of bioactive compounds that can be extracted from natural sources, lupane-type triterpenoids such as betulin (Bet, lup-20(29)-ene-3β,28-diol) and betulinic acid (BAc, 3β-hydroxy-20(19)-lupaen-28-oic acid) have been the subject of extensive research because of their wide variety of biological and pharmacological activities (anti-inflammatory, antibacterial, antimalarial, antidiabetic, anti-HIV, anticancer, etc.) [7–9]. These pentacyclic lupane-type triterpenoids are widely distributed throughout the plant kingdom [8, 10] and can be isolated, among others, from forest byproducts such as birch (*Betula* sp.) bark (BB) [7, 8].

Whereas the improved liquid chromatography-mass spectrometry (LC-MS) analysis of Bet and BAc has recently been optimized and validated [11], there is a demand for the development of greener and improved procedures for the simultaneous extraction of these two BB bioactive compounds.

Although Soxhlet and solid-liquid extraction (SLE) using numerous conventional organic solvents (e.g. methanol, chloroform, dichloromethane, etc.) have been traditionally employed for their extraction [12–15], increasing attention has recently been given to the evaluation of alternative greener extractants such as natural deep eutectic solvents (NADESs) [10].

NADESs are mixtures of natural hydrogen bond donor (HBD) and hydrogen bond acceptor (HBA) species, whose melting points are lower than those of single components and far lower than the ambient temperature [16]. Among other advantages over volatile organic solvents, NADESs are typically considered less toxic, biodegradable, cost-efficient and easier to produce and can be tailor-designed. All these features make them promising extractants for the development of ecosustainable approaches. Despite the relatively large number of applications involving the use of hydrophilic NADESs for the extraction of target bioactives [16–19], studies that address the use of hydrophobic NADESs (h-NADESs) are much more limited, notwithstanding their potential for the extraction of numerous nonpolar high-value bioactives [20–22]. Moreover, h-NADESs have scarcely been exploited for the extraction of bioactive triterpenoids [10], and no study has investigated their application for the simultaneous extraction of both Bet and BAc from birch bark.

Given the large number of potential combinations of HBD and HBA candidates, the experimental selection of the most suitable h-NADES to be used as an extractant of BB bioactives can be not only a tedious and time-consuming task but also an expensive procedure. To overcome these issues, the use of predictive approaches such as the COnductor-like Screening MOdel for Real Solvents (COSMO-RS) is generally preferred [23].

On the other hand, enhanced extraction techniques such as pressurized liquid extraction (PLE) [24–27], microwave-assisted extraction (MWAE) [15, 28, 29], ultrasound-assisted extraction (UAE) [30–33], etc. have been described to provide advantages over conventional SLE methods for the improved (i.e. faster, with increased yield and/or selectivity, etc.) recovery of BB bioactives. However, the majority of these studies have focused on the extraction of individual BB bioactives (mainly Bet), and none has addressed the use of UAE in combination with h-NADESs for the efficient simultaneous extraction of Bet and BAc. Moreover, the assessment of the potential advantages in terms of the sustainability of these novel sample preparation approaches via greenness metrics such as AGREEprep [34] is increasingly in demand.

At the sight of these antecedents, the main objectives of this paper are (i) the *in silico* selection by the COSMO-RS theory of the optimal extractant of lupane-type BB bioactives (Bet and BAc) among numerous conventional organic solvents, biosolvents and h-NADESs; (ii) the optimization (by means of an experimental design), analytical characterization and greenness evaluation of an efficient and sustainable h-NADES UAE approach for the simultaneous recovery of these two high-value triterpenoids and (iii) the application of this novel method to different *Betula* sp. bark samples.

## Materials and methods

### Standards and samples

Analytical standards of Bet and BAc (> 99% purity) were acquired from Sigma-Aldrich (St. Louis, MO, USA). Six birch bark samples were purchased from different suppliers (BB1: *Betula* sp.; BB2 and BB4–BB6: *B. pendula*; BB3: *B. alba*). All BB samples were ground to fine particles with an IKA A10 basic mill (IKA-Werke, Germany), sieved through a 500 µm mesh and stored in amber vials at room temperature until extraction.

### Computational methodology for solvent screening

*In silico* solvent screening requires prior optimization of the three-dimensional geometry of the target molecules. In the present work, this optimization was performed using the Turbomole 7.3 software package with the TmoleX 4.4.1 graphical user interface (3DS Biovia, Vélizy-Villacoublay, France). The optimal molecular structures were calculated from their smiles codes using density functional theory (DFT) at the BP86 functional level and a triple zeta valence polarization (TZVP) basis set. The results were saved as *.cosmo* files and further used for COSMO-RS calculations using the COSMOThermX 21.0 software package (3DS Biovia) with the parametrization BP_TZVP_21.ctd. Sigma surfaces, sigma profiles and sigma potentials of Bet, BAc and solvents (conventional organic solvents, terpenoids and h-NADESs) were calculated. The natural logarithms of the infinite dilution activity coefficients (${\mathrm{ln}(\gamma }_{i}^{\infty })$) of Bet and BAc in the selected solvents were estimated. The temperature and molar ratios of the eutectic mixtures were also considered in the calculations.

The activity coefficients are considered suitable indicators of intermolecular interactions between a solvent and any solute *i* and, therefore, quantitative descriptors of the solvation power of a solvent. When the ${\gamma }_{i}^{\infty }$ value for a given solute–solvent pair is less than unity, the net interactions are attractive, the solvation process is favoured, and the solvent might be considered a potential candidate for analyte extraction. In contrast, if the value is very high, solute-solvent interactions are weak, and the solvent is not appropriate. ${\gamma }_{i}^{\infty }$ is also used to calculate the solvent capacity at infinite dilution (${C}_{i}^{\infty }$) (Eq. 1), which is a measure of the dissolution ability of a solvent for a specific solute (*i*).

1 $${C}_{i}^{\infty }=\frac{1}{{\gamma }_{i}^{\infty }}$$

### Preparation and characterization of hydrophobic natural deep eutectic solvents

The reagents used in the preparation of the selected h-NADES, all with a purity > 98%, were thymol (Thermo Fisher Scientific, Germany) as the HBD and anhydrous 1-octanol (Sigma-Aldrich) as the HBA. HBD:HBA mixtures (molar ratio of 4:1) were heated on a heating plate provided with a temperature probe (IKA RCT basic, IKA Labortechnik, Germany) at 60 °C for 1 h under stirring conditions (600 rpm) to obtain a homogenous colourless liquid h-NADES.

 Differential scanning calorimetry (DSC) analysis of this h-NADES was carried out using a Q100 calorimeter (TA Instruments, New Castle, DE, USA) connected to a cooling system. The temperature evaluated ranged from −55 to 55 °C at a scan speed of 5 °C min^−1^ after a cooling-heating cycle. 

The viscosity (in mPa·s) and density (in g mL^−1^) of this h-NADES were measured in triplicate at 25 °C with an SVM 3000 viscometer (Anton Paar, Austria) to estimate its feasibility as an extractant.

### Extraction of birch bark bioactives

#### Solid-liquid extraction (SLE)

SLE assays were performed using a heating plate (IKA RCT basic) equipped with temperature and stirring control. Ground BB samples (100 mg) were mixed with 1 mL of the h-NADES previously prepared (“Preparation and characterization of hydrophobic natural deep eutectic solvents”) and further extracted for 30 min at different temperatures (25, 55 and 75 °C) under stirring conditions (600 rpm). The extracts were further centrifuged at 4400 × *g* for 10 min and filtered through 0.2 µm polytetrafluoroethylene (PTFE) membranes (Interchim, Montluçon, France) prior to LC-MS analysis. All extractions were performed in triplicate.

#### Ultrasound-assisted extraction (UAE)

UAE experiments were carried out in an ultrasonic bath (Elma Schmidbauer GmbH, Singen, Germany) (300 × 151 × 150 mm; tank service capacity of 5.75 L) operating at a frequency of 37 kHz and 30% power to provide a stable temperature (± 2 °C) throughout the entire extraction process. First, a set of preliminary experiments was carried out to compare the extraction performance of SLE with that of UAE under identical temperature and time conditions (100 mg BB: 1 mL h-NADES; 55 °C for 30 min).

On the basis of the conclusions drawn from these preliminary experiments, in-depth optimization of the UAE was performed via a face-centred central composite (2^2^ + star) experimental design. The effects of two independent factors (temperature (*T*, °C) and time (*t*, min)) on the simultaneous extraction of Bet and BAc were explored in 11 randomized runs. The experimental ranges for the factors evaluated were *T* = 35–75 °C and *t* = 5–30 min (Table S1 of the Supplementary Material). The proposed quadratic model is expressed as follows:

2 $$R\:=\:\beta_0\:+\:\beta_1T\:+\:\beta_2t\:+\:\beta_{1,1}T^2\:+\:\beta_{2,2}t^2\:+\:\beta_{1,2}\;T\cdot t\:+\:\varepsilon\;$$

where *β*_0_ represents the intercept, *β*_*i*_ denotes the first-order coefficients, *β*_*i*,*i*_ indicates the quadratic coefficients for the *i*th factors, *β*_*i*,*j*_ represents the coefficient for the interaction of factors *i* and *j*, and *ε* is the error. Two response variables were considered in the optimization of the UAE method: Bet concentration (*R*_Bet_, mg g^−1^ sample) and BAc content (*R*_BAc_, mg g^−1^ sample). The experimental UAE parameters individually maximizing *R*_Bet_ and *R*_BAc_ were estimated by multiple linear regression using StatGraphics Centurion XVI software (Statistical Graphics Corporation, Rockville, MD, USA). A desirability function (*R*_D_) was also calculated to provide the UAE conditions that simultaneously maximized both responses. This *R*_D_ function takes values between 0 (completely undesirable value) and 1 (completely desirable or ideal response).

To fully optimize the UAE method, the effect of the number of cycles (C1–C3) on the bioactive extraction yield was also evaluated (*n* = 3). Moreover, to shorten the whole extraction process, and in accordance with a previous study [35], the simultaneous preparation of the h-NADES selected as optimal in “Computational methodology for solvent screening” and the extraction of BB bioactives were also assayed (*n* = 3) under the UAE operating conditions previously optimized. All the extracts mentioned above were further processed as described in “Solid-liquid extraction (SLE)” prior to their LC-MS analysis.

### LC-MS analysis

The intended analysis of the concentrations of BB bioactives from h-NADES extracts was performed as previously described by Luque-Jurado et al. [11]. LC-MS analyses were performed on a 1260 Infinity II Prime LC system, equipped with an autosampler, quaternary pump, column heater compartment and diode array detector, coupled via an electrospray ionization (ESI) source to a 6125 single quadrupole mass spectrometry detector (both from Agilent Technologies, Santa Clara, CA, USA). Separations were carried out using a Poroshell 120 EC-C18 column (150 × 3 mm, 2.7 μm; Agilent Technologies) thermostated at 35 °C. A mobile phase consisting of (A) water + 0.1% formic acid (Thermo Fisher Scientific), (B) 1 mM sodium acetate (Sigma-Aldrich) and (C) methanol was used for the resolution of the target bioactives and for stabilization of their MS response, according to the following program: 0–25 min: 85% A/1% B/14% C; 26–31 min: 1% A/1% B/98% C; and 32–41 min: back to the initial conditions. The injection volume and flow rate were 10 μL and 0.3 mL min^−1^, respectively.

The ESI parameters for MS detection were as follows: fragmentor voltage (160 V for Bet and 300 V for BAc), nebulizing gas (35 psig), capillary voltage (4000 V), and drying gas flow and temperature (12 L min^−1^ and 350 °C).

Compound identification was based on chromatographic retention data and full scan mass spectra (100–1000 *m*/*z* range) and was confirmed by coinjection of the corresponding commercial standards. Quantitation (*n* = 3) of h-NADES extracts, diluted 1:133 (*v/v*) with methanol (J.T. Baker, MA, USA) to avoid matrix effects, was performed via the external standard method, employing data acquired in single-ion monitoring (SIM) mode: [M + Na]^+^  = 465 for Bet and [M-H]^−^ = 455 for BAc. Standard solutions in the ranges of 0.0002–0.1 mg mL^−1^ and 0.0001–0.1 mg mL^−1^ were prepared for the Bet and BAc calibration curves, respectively. Data acquisition and processing were performed using OpenLAB CDS Software (v.2.19.20, Agilent Technologies). The results are expressed as mg g^−1^ sample.

### Analytical characterization of the h-NADES UAE method

The reproducibility of the optimized h-NADES UAE method, determined by LC-MS [11] analysis, was measured in terms of intra- and interday precision by analysing the Bet and BAc concentrations of the BB1 extract within the same day (*n* = 5) or on five consecutive days, respectively. Recovery (%) was calculated by spiking the BB1 sample with standard solutions at three concentrations (20–80 µL of 0.01 mg mL^−1^ Bet and 60–80 µL of 0.003 mg mL^−1^ BAc) and further extraction. All the assays were performed in triplicate.

### Sustainability assessment by the AGREEprep metric

The greenness of the extraction method developed in this study by h-NADES UAE, including the required LC-MS characterization of the composition of the extracts, was carried out with the AGREEprep metric [34]. After the weights for each of the ten green sample preparation (GSP) principles are assessed, a pictogram with a greenness overall score in the 0–1 range (the closer to 1, the greener the method is) is constructed. Moreover, criteria that better fulfil (green) or do not fulfil (red) GSP principles are also identified.

### Statistical analysis

Statgraphics Centurion XVI software (Statgraphics Technologies, Inc.) was used for statistical analysis of the data. The significance (*p* < 0.05) of differences for extractions that were carried out with different techniques and obtained under different UAE operating conditions or from different BB samples was determined by analysis of variance (ANOVA, Tukey test).

## Results and discussion

### *In silico* solvent selection for the extraction of BB bioactives

**Fig. 1:**
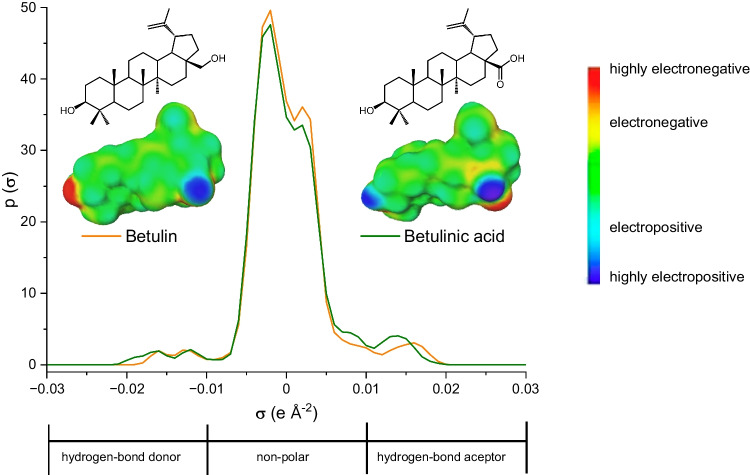
Chemical structures, sigma surfaces and sigma profiles of betulin and betulinic acid estimated by COSMO-RS

The simultaneous extraction of both Bet and BAc from birch bark was evaluated* in silico *for the first time in this paper on the basis of their potential interactions estimated via COSMO-RS theory. Thus, the sigma profiles of both triterpenoids (Fig. 1) revealed that they are able to establish not only apolar interactions (–0.01 < *σ* < 0.01), but they are also capable of acting as hydrogen bond donors and acceptors, as shown by the peaks at *σ* < –0.01 and *σ* >  + 0.01, respectively. These common characteristics make their simultaneous extraction feasible under similar operating conditions.

**Fig. 2:**
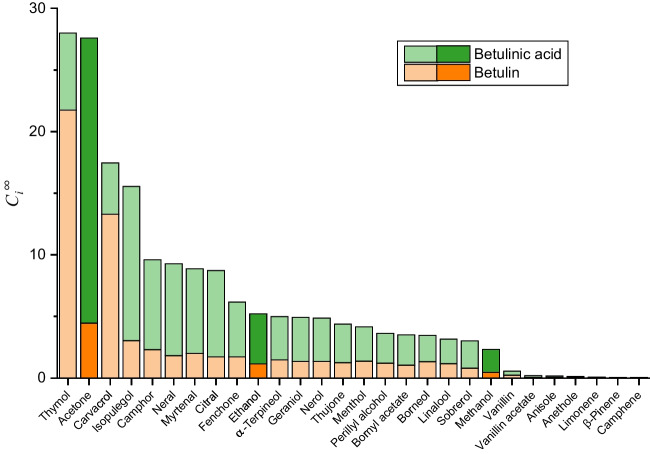
Infinite dilution solvent capacity (${C}_{i}^{\infty }$) of solvents under study estimated by COSMO-RS for Bet and BAc at 25 °C. Terpenoids and conventional organic solvents are shown as pale-colour and dark-colour bars, respectively

Taking into account the information provided by COSMO-RS, the use of a single h-NADES for the simultaneous recovery of Bet and BAc was considered. To select the HBD/HBA candidates with the best extraction performance, the individual solvent capacity of 25 terpenoids for these bioactives was evaluated. Monoterpenes such as limonene, which were previously described for the extraction of triterpenoic acids [10], as well as numerous terpenoids (e.g. menthol, thymol, camphor, etc.) reported as components of h-NADESs exhibiting improved extraction of plant and food bioactives [10, 21], were included in this study. Moreover, for comparison purposes, conventional organic solvents that were previously reported for the extraction of these compounds (e.g. methanol, ethanol and acetone) were also considered (Fig. 2).

Thymol and carvacrol provided the highest ${C}_{i}^{\infty }$ for Bet (${C}_{\mathrm{Bet}}^{\infty }$, 21.7 and 13.3, respectively); these values were also significantly higher than those provided by other terpenoids or conventional solvents. With respect to BAc, the ${C}_{\mathrm{BAc}}^{\infty }$ values were higher for acetone and isopulegol (23.1 and 12.5, respectively), with thymol (among other solvents) yielding intermediate results (${C}_{\mathrm{BAc}}^{\infty }$= 6.2). Considering the intended simultaneous extraction of both Bet and BAc, the sum of the solvent capacity for Bet and BAc (${C}_{\mathrm{Bet}+\mathrm{BAc}}^{\infty }$) was chosen as the criterion for selecting the optimal solvent. As shown in Fig. 2, thymol was the compound providing the highest ${C}_{\mathrm{Bet}+\mathrm{BAc}}^{\infty }$ value; therefore, it was selected as the first component of the h-NADES to be used in this study.

**Fig. 3:**
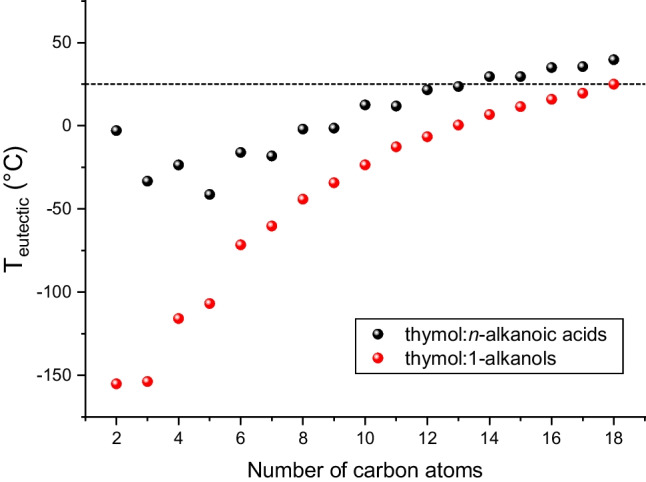
Variation of estimated eutectic temperatures (*T*_eutectic_, °C) with the number of carbon atoms of *n*-alkanoic acids and 1-alkanols used as the second component of thymol-based h-NADES (1:1 molar ratio)

Because thymol mainly acts as a HBD, compounds such as alcohols and carboxylic acids were considered in the selection of the second component (HBA) of thymol-based h-NADESs. Eutectic mixtures of thymol and the different members of the homologous series of 1-alkanols and *n*-alkanoic acids were simulated to estimate the corresponding eutectic temperature (*T*_eutectic_). As shown in Fig. 3, the mixtures including alcohols had lower estimated melting temperatures than those obtained with acids did. Therefore, the former (specifically from ethanol to 1-hexadecanol) were chosen for additional assays to ensure that the h-NADESs to be prepared were liquid at room temperature and that no additional heating was needed. This energy savings would also positively contribute to the development of greener methods for BB bioactive extraction.

**Fig. 4:**
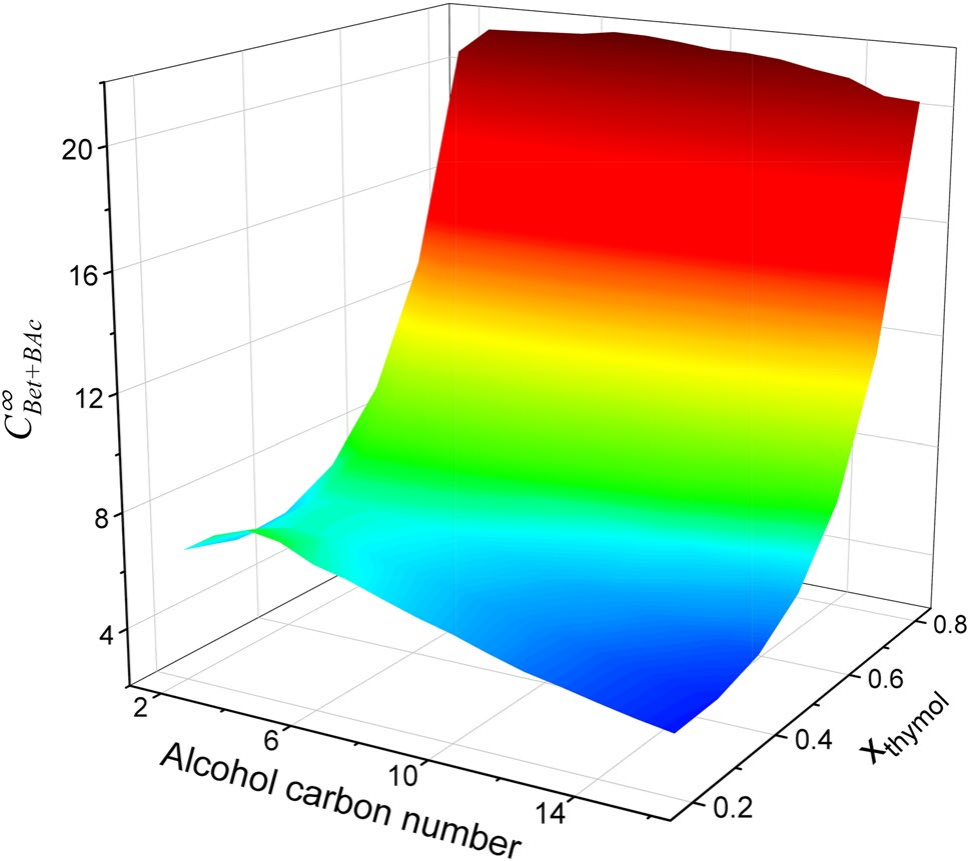
Effect of the thymol:1-alkanol molar fraction (*x*_thymol_, from 0.2 to 0.8) and the chain length of the 1-alkanol on ${C}_{\mathrm{Bet}+\mathrm{BAc}}^{\infty }$ at 25 °C predicted by COSMO-RS

The last calculation step involved estimation of the ${C}_{\mathrm{Bet}+\mathrm{BAc}}^{\infty }$ values of the thymol:1-alkanol (from ethanol to 1-hexadecanol) mixtures with different HBD:HBA molar fraction (*x*_thymol_, from 0.2 to 0.8) at 25 °C. As shown in Fig. 4, a molar ratio of 4:1 (*x*_thymol_ = 0.8) resulted in the highest solvent capacity for all the alcohols that were tested (${C}_{\mathrm{Bet}+\mathrm{BAc}}^{\infty }$= 20.3–21.7). With respect to the number of carbon atoms, the highest ${C}_{\mathrm{Bet}+\mathrm{BAc}}^{\infty }$ value was obtained for C_7_–C_8_ alcohols. Considering that low volatility and low hydrophilicity are desirable characteristics for obtaining green hydrophobic NADESs, the thymol:1-octanol (4:1) mixture was chosen as the optimal extractant of Bet and BAc (${C}_{\mathrm{Bet}+\mathrm{BAc}}^{\infty }$ = 21.7).

**Fig. 5:**
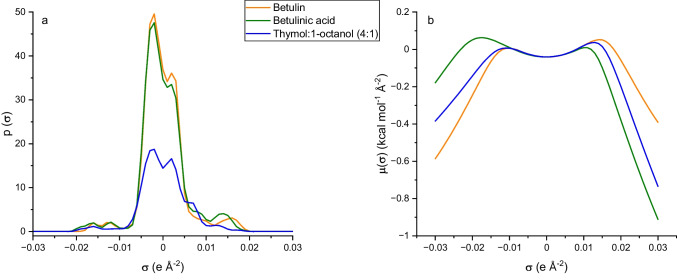
Sigma profiles (**a**) and sigma potentials (**b**) of betulin, betulinic acid and thymol:1-octanol (4:1) h-NADES estimated by COSMO-RS

The sigma profiles and sigma potentials for thymol:1-octanol (4:1) h-NADES, betulin, and betulinic acid are shown in Fig. 5. The shape of the sigma potentials allows us to rationalize the expected ability of the selected h-NADES to easily coextract both bioactive compounds. There is a perfect match in the region of −0.01 < *σ* < 0.01, indicating favourable hydrophobic and electrostatic interactions. Moreover, this h-NADES has a good capacity to interact through donor (*σ* < −0.01) and acceptor (*σ* >  + 0.01) hydrogen bonds because it presents strong negative sigma potential values in both zones. Notably, the calculations performed adequately modulated the intensity of the interactions between the selected h-NADES and the two bioactives to be extracted, because the descending branches of the sigma potential in the hydrogen bond donor/acceptor zones of thymol:1-octanol (4:1) are between those of the two bioactives irrespective of the zone considered. This finding indicates a weighted interaction intensity for both compounds, which presumably can result in their good coextraction. On the basis of the excellent results obtained in this simulation study, thymol:1-octanol (4:1) h-NADES was used in the laboratory to develop a green and efficient method for the extraction of birch bark target bioactives.

### Simultaneous extraction of BB bioactives

#### Experimental evaluation of thymol:1-octanol (4:1) h-NADES as an extractant

Prior to the use of thymol:1-octanol (4:1) as an extractant of BB bioactive compounds, the thermal behaviour of this h-NADES was evaluated by DSC. The DSC thermogram (Fig. S1) clearly shows a melting point depression, with an onset temperature (*T*_m, onset_) of 23.1 °C, which is below the melting point of thymol (51.5 °C) and significantly lower than the estimated ideal eutectic mixture temperature (*T*_m, ideal_ = 40.7 °C). This thermal profile confirms the formation of a stable liquid phase at working temperatures, which fulfills the functional criteria for classification as a deep eutectic solvent [36] and supports its use as a BB bioactive extractant.

Moreover, this h-NADES was physicochemically characterized in terms of density and viscosity to confirm its feasibility of application. Compared with other high-density hydrophilic NADESs for the extraction of plant bioactives [17] and different terpenoid-based h-NADESs [37], thymol:1-octanol (4:1) was characterized by a noticeably lower density (0.94 g cm^−3^) and viscosity (12.4 mPa·s). From an application point of view, these properties not only favour the extraction process but also contribute to improving its sustainability because simpler facilities and a lower energy demand are required when low-density and low-viscosity solvents are used as extractants.

#### SLE vs. UAE

To evaluate the experimental performance in terms of the extraction yield (mg g^−1^ sample) of the h-NADES previously selected in “*In silico* solvent selection for the extraction of BB bioactives”, SLE assays that use thymol:1-octanol (4:1) as the extractant were carried out under different temperature conditions (25 and 55 °C). As shown in Table 1, the extraction yield of both bioactives significantly increased with temperature, with values that were 79% and 39% greater at 55 °C for Bet and BAc, respectively. In agreement with these results, an improved extraction yield of triterpenoid acids such as BAc has been reported when conventional extractions (maceration, stirring, etc.) with either organic solvents (ethanol, CH_2_Cl_2_, etc.) or h-NADESs (e.g. menthol:thymol combinations at different molar ratios) are carried out at temperatures above ambient temperature [10].

**Table 1:** Concentration (average and standard deviation in brackets for *n* = 3) of betulin (Bet) and betulinic acid (BAc) recovered from sample BB1 by solid-liquid extraction (SLE) or ultrasound-assisted extraction (UAE) under different temperature conditions

Technique	*T* (°C)	*t* (min)	Bet (mg g^−1^)	BAc (mg g^−1^)
SLE	25	30	11.41 (0.34)^a,*^	0.56 (0.01)^a^
SLE	55	30	20.43 (0.63)^b^	0.78 (0.02)^b^
UAE	55	30	25.08 (2.13)^c^	1.00 (0.03)^c^

^*^Superscripts ^a–c^ within the same column indicate statistically significant differences for mean values at the 95% confidence level

The effect of the extraction technique was further evaluated by a comparison of SLE and UAE under identical extraction conditions (55 °C for 30 min). Compared with SLE, the use of an additional source of energy (US) in UAE improved the extraction yields of Bet (31%) and BAc (28%) (Table 1). These results are consistent with those of previous studies, which revealed that UAE, combined with organic solvents, outperforms (in terms of efficiency and speed) conventional techniques such as maceration for the extraction of birch triterpenoids [31, 32].

#### Development and analytical characterization of a green and efficient h-NADES UAE method for the simultaneous extraction of BB bioactives

A face-centred central composite experimental design was considered for the in-depth optimization of UAE operating conditions (time and temperature) in the ranges previously described in Table S1. In general, UAE experiments at low temperature (35 °C) or for short extraction times (up to 17.5 min) resulted in the lowest extraction of both bioactive compounds (Bet: 14.87–19.08 mg g^−1^; BAc: 0.61–0.78 mg g^−1^). Although an increased recovery of these two lupane-type triterpenoids was found when higher extraction temperatures were used (≥ 55 °C) (up to 26.78 mg g^−1^ Bet and 1.00 mg g^−1^ BAc), a significant degradation of both bioactives was detected at 75 °C, particularly when the extraction was extended for long times (down to 19.99 mg g^−1^ Bet and 0.89 mg g^−1^ BAc, respectively).

Response surface methodology was further used to calculate the regression coefficients of the models for the Bet and BAc responses (*R*_Bet_ and *R*_BAc_, respectively) and their statistical significance, as well as to estimate the prediction errors (standard error (SE) and mean absolute error (MAE)). The experimental conditions that individually maximized *R*_Bet_ and *R*_BAc_ were also obtained (Table S2, Fig. S2). As indicated by the Pareto diagrams (Fig. S3), time (*t*) was the only significant factor at the 95% confidence level (*p* < 0.05) for both responses, and similar extraction temperature and time conditions were shown to be optimal for the individual extraction of either Bet or BAc (59 °C for 26 min and 62 °C for 22 min, respectively). With respect to the SE and MAE errors, the variability explained by the corresponding models was high enough to ensure the accuracy of the prediction. Moreover, when a multiple response (*R*_D_ = 0.88) that simultaneously maximized *R*_Bet_ and *R*_BAc_ was considered, 61 °C and 24 min were selected as the optimal UAE operating parameters to provide BB extracts rich in bioactives (25.17 mg g^−1^ Bet and 1.06 mg g^−1^ BAc, respectively). Under these conditions, a good match (relative error < 2%) between the experimental and predicted amounts of Bet and BAc recovered by UAE was obtained.

The number of extraction cycles of the optimized h-NADES UAE method was subsequently evaluated. As the percentage of both bioactives decreased similarly in cycles C1–C3 (87 > 9 > 4% for Bet and 85 > 10 > 5% for BAc), a single cycle was considered a trade-off to provide a high-throughput extraction method with enough recovery of both bioactive triterpenoids.

With respect to the development of not only an efficient UAE method based on the use of green solvents but also a sustainable approach reducing the energy demand to a minimum, the possibility of shortening the overall extraction process by considering the simultaneous preparation of the h-NADES thymol:1-octanol (4:1) and the UAE extraction of BB bioactives was considered under the UAE conditions previously optimized. However, the experimental recoveries of Bet and BAc via this joint approach were lower (15 and 13%, respectively) than those of the conventional two-step procedure. Therefore, this fully integrated method was discarded for applications in which maximization of the recovery of BB bioactives is intended, like in the present research.

The analytical characterization of the h-NADES UAE approach developed in this study was carried out by the LC-MS method previously optimized and validated for the analysis of Bet and BAc in biosolvent extracts, including the h-NADES thymol:1-octanol used here as an extractant [11].

As previously reported, no interference arising from the coelution of h-NADES components with target bioactives was detected by LC-MS analysis of these BB extracts (Fig. S4). The linearity of this LC-MS method in terms of the concentration ranges encompassing the concentrations of Bet and BAc in h-NADES UAE extracts and well above the detection limits of this separation method (23 and 29 ng mL^−1^ for Bet and BAc, respectively) was also determined (Bet: *y* = 2.68·10^8^ *x* − 573,712, *R*^2^ = 0.992; BAc: *y* = 2.39·10^8^ *x* + 27,502, *R*^2^ = 0.997).

The figures of merit regarding the reproducibility and recovery of this new extraction method are shown in Table S3. On the basis of intraday and interday precision data (RSD ≤ 1.60% for Bet and RSD ≤ 2.27% for BAc) and recoveries estimated after the BB1 sample was spiked at three concentrations (≥ 96.22% and 79.88% for Bet and BAc, respectively), a precise and high enough accurate extraction of both BB bioactives was achieved via the h-NADES UAE methodology developed in the present study.

**Fig. 6:**
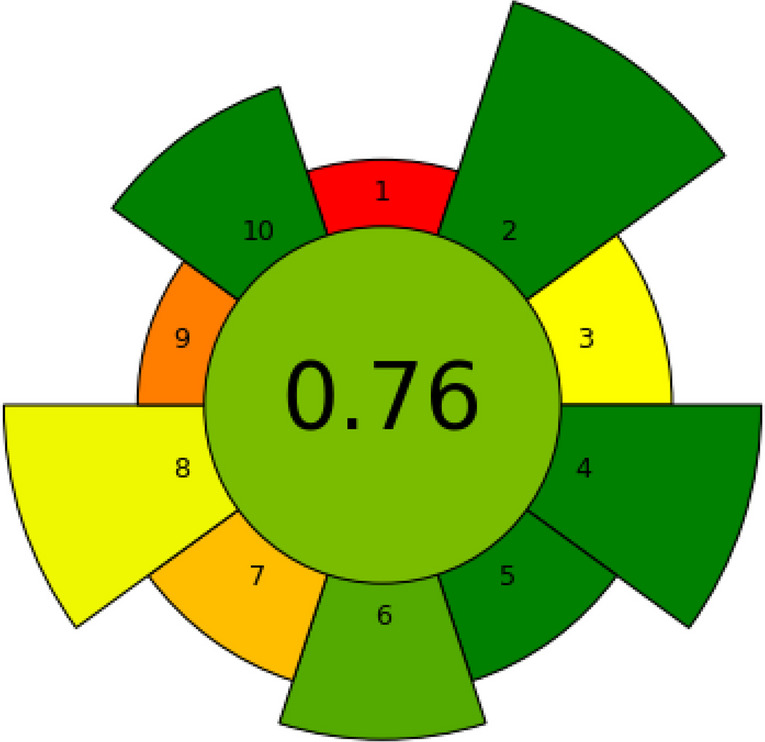
AGREEprep pictogram with the overall greenness score obtained in the sustainability assessment of the optimised h-NADES UAE method

The quantitative sustainability assessment of this method was further evaluated by the AGREEprep metric [34]. Table S4 lists the assigned weights and individual scores estimated for each of the GSP principles, whereas the overall greenness score obtained by this method is presented in the pictogram of Fig. 6. The use of nonhazardous materials such as h-NADESs and the low volume of waste that is generated (factors 2 and 4), the minimal amount of a forestry byproduct rich in bioactives (factor 5) and operator safety (factor 10) were the main positive contributions to the AGREEprep overall score (0.76). The inevitable ex situ sample preparation placement was, in contrast, the most negative contribution.

### Application of the optimized h-NADES UAE method to different BB samples

Table 2 summarizes the results obtained after the application of the h-NADES UAE method previously optimized for the simultaneous extraction of Bet and BAc from samples BB1–BB6. A wide variability in the concentrations of BB bioactive compounds was observed for the samples considered in this study. Although the UAE extract of BB5 was richer in both bioactives (32.17 mg Bet g^−1^ and 1.43 mg BAc g^−1^), the content of these lupane-type triterpenoids was not fully dependent on the species considered, because the concentrations of Bet and BAc were significantly lower in the other *B. pendula* extracts.

**Table 2:** Concentration of betulin (Bet) and betulinic acid (BAc) (average and standard deviation in brackets for *n* = 3) in the h-NADES UAE extracts obtained under optimal conditions (thymol:1-octanol (4:1), 61 °C for 24 min, 1 cycle) from birch bark samples under study

Sample	Bet (mg g^−1^)	BAc (mg g^−1^)
BB1	25.17 (0.12)^e,*^	1.06 (0.001)^d^
BB2	21.93 (0.10)^d^	0.96 (0.002)^c^
BB3	16.08 (0.58)^c^	0.82 (0.03)^b^
BB4	13.38 (0.61)^b^	0.72 (0.05)^a^
BB5	32.17 (0.33)^f^	1.43 (0.06)^e^
BB6	12.09 (0.17)^a^	0.69 (0.02)^a^

^*^Superscripts ^a–f^ within the same column indicate statistically significant differences for mean values at the 95% confidence level

As previously described [12, 15, 27, 29], the concentration of bioactive triterpenoids in BB extracts is affected not only by the extraction procedure and conditions that were employed but also by many other factors, including *Betula* species, provenance and growth conditions, age of the tree and tissue considered, etc. Thus, contents of BAc (2.8 mg g^−1^ sample, *Betula* species not detailed) and Bet (in the range 20–30 mg g^−1^ depending on the type of birch bark) similar to those of the present study have been reported in the application of other advanced extraction techniques, such as subcritical water extraction (SWE, 27 min, 184 °C) [26] or ethanolic PLE (15 min, 120 °C) [24]. Although in both cases the extraction time was similar to that of the h-NADES UAE method here developed (24 min), the use of a noticeably lower extraction temperature (61 °C) in the present work would support its higher sustainability.

Considering the limited number of studies that address the use of h-NADESs for the extraction of bioactive triterpenoids, a lower extraction yield of BAc (0.41 mg g^−1^) and a much longer extraction time (4 h) have been reported in the SLE of *Eucalyptus globulus* biomass at 90 °C when menthol:thymol (molar ratio of 1:2) was used as the extractant [10]. These results support the previously mentioned requirement of careful selection of the most appropriate biomass and the optimal extraction approach when extracts rich in bioactive triterpenoids are intended.

## Conclusions

In this study, which aimed to develop an efficient and sustainable extraction method for the simultaneous recovery of birch bark bioactives for the first time, the use of the COSMO-RS theory allowed the *in silico* selection of thymol:1-octanol (4:1) as the optimal extractant of both Bet and BAc.

The optimization, via an experimental design, of this h-NADES UAE method resulted in an improved extraction yield of BB lupane-type triterpenoids over the SLE approaches conventionally used for this purpose. Moreover, the good precision and accuracy, as well as the sustainability score assessed by the AGREEPrep metric, confirm that this method is useful for obtaining green extracts rich in BB triterpenoids.

Although the application of this optimized methodology to BB samples has demonstrated the potential of this forestry byproduct as a natural source of high-value bioactives, the selection of the biomass is still key when BB extracts with bioactive properties are intended.

Although the feasibility of scaling up this h-NADES UAE methodology should be considered advantageous for its application at the industrial scale in different fields (food science, cosmetics, pharmacy, etc.), further research is needed to provide efficient and cost-effective approaches for solvent removal (if dry BB extracts are required) and for the recovery and reuse of h-NADESs if an ecosustainable strategy for fulfilling the White Analytical Chemistry principles is to be achieved.

## Supplementary Information

Below is the link to the electronic supplementary material.

Supplementary Material 1 (DOCX 175 KB)

## Data Availability

All data are included in the main body or in the Supplementary Material of the paper.
