# Supplementary material for: Ultrasound-assisted extraction of bioactive birch (Betula sp.) bark triterpenoids using hydrophobic natural deep eutectic solvents
Source: Anal Bioanal Chem. 2025 Nov 27;418(6):1713–23. doi: 10.1007/s00216-025-06174-7 (PMC12960316; doi:10.1007/s00216-025-06174-7)
Supplement: Supplementary file 1 — Supplementary Material 1 (DOCX 175 KB) [file 216_2025_6174_MOESM1_ESM.docx]

**SUPPLEMENTARY MATERIAL**

**ULTRASOUND-ASSISTED EXTRACTION OF BIOACTIVE BIRCH (*Betula* sp.) BARK TRITERPENOIDS USING HYDROPHOBIC NATURAL DEEP EUTECTIC SOLVENTS**

I. Luque-Jurado^1^, S. Rivas^1^, R. Lebrón-Aguilar^2^, J.E. Quintanilla-López^2^, M.L. Sanz^1^, A.C. Soria^1,*^


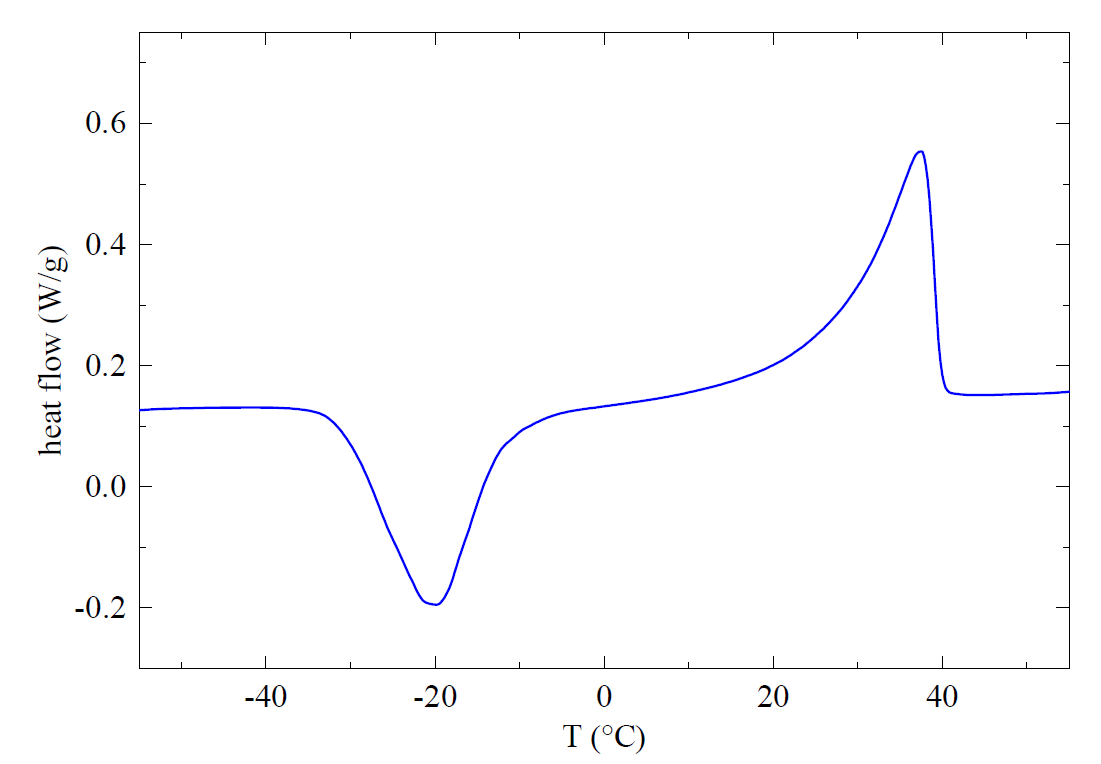


**Fig. S1** Differential Scanning Calorimetry (DSC) analysis of h-NADES thymol:1-octanol (4:1 molar ratio)

**Fig. S2** Response surfaces for (A) Bet and (B) BAc obtained in the optimization of h-NADES UAE operating conditions (Temperature (*T*) and time (*t*)) by means of a central composite experimental design

**Fig. S3** Standardized Pareto diagrams for Bet and BAc obtained in the optimization of h-NADES UAE operating conditions (Temperature (*T*) and time (*t*)) by means of a central composite experimental design

**Fig. S4** LC-MS chromatographic profile of the h-NADES UAE extract from BB1 sample and of Bet and BAc standards under experimental conditions described in [11]: 0-25 min: 14 % H_2_O + 0.1 % formic acid (A) / 1 % sodium acetate 1 mM (B) / 85 % methanol (C); 26-31 min: 1 % A / 1 % B / 98 % C; 32-41 min: back to initial conditions

**Table S1** Results of the central composite experimental design for optimization of h-NADES UAE of birch bark bioactives: betulin (Bet) and betulinic acid (BAc)

| ***T* (°C)** | ***t* (min)** | **Bet**  **(mg g^-1^)** | **BAc**  **(mg g^-1^)** |
| --- | --- | --- | --- |
| 35 | 5 | 16.06 | 0.68 |
| 55 | 17.5 | 23.78 | 1.09 |
| 55 | 17.5 | 23.21 | 0.99 |
| 55 | 17.5 | 24.46 | 1.05 |
| 75 | 30 | 19.99 | 0.89 |
| 55 | 30 | 26.78 | 1.00 |
| 35 | 17.5 | 15.86 | 0.70 |
| 75 | 5 | 16.13 | 0.69 |
| 55 | 5 | 14.87 | 0.61 |
| 75 | 17.5 | 23.39 | 1.01 |
| 35 | 30 | 19.08 | 0.78 |

**Table S2** Summary of the results obtained in the optimization by means of a central composite experimental design of the UAE operating conditions providing maximal recovery of BB bioactives

| *R_Bet_* | Model equation:  *R_Bet_* = -15.55 + 1.04·*T* + 0.74·*t* – 0.0089·*T*^2^ + 0.00084·*Tt* – 0.015·*t*^2^  (R^2^ = 76.02 %) |
| --- | --- |
|  | Error: SE = 2.214; MAE = 1.211 |
|  | Optimal conditions: 59 °C, 26 min |
|  | Predicted response under optimal conditions: 24.77 mg g^-1^ |
| *R_BAc_* | Model equation:  *R_BAc_* = -0.42 + 0.033·*T* + 0.040·*t* – 0.00028·*T*^2^ + 0.0001·*Tt* – 0.0010·*t*^2^  (R^2^ = 80.94 %) |
|  | Error: SE = 0.080; MAE = 0.045 |
|  | Optimal conditions: 62 °C, 22 min |
|  | Predicted response under optimal conditions: 1.05 mg g^-1^ |

**Table S3** Analytical characterization of the h-NADES UAE method developed for extraction of BB bioactives, following LC-MS analysis [11]

|  | | **Bet** | **BAc** |
| --- | --- | --- | --- |
| Intraday precision  (RSD %, *n* =5) | | 0.73 | 0.49 |
| Interday precisión  (RSD %, *n* =5) | | 1.60 | 2.27 |
| Accuracy (%)* | | **Bet** | **BAc** |
| ng Bet | ng BAc |  |  |
| 225 | 180 | 96.22 (1.10) | 82.92 (7.80) |
| 450 | 210 | 99.66 (1.16) | 79.88 (5.34) |
| 900 | 240 | 99.17 (1.35) | 80.54 (1.63) |

*Standard deviation into brackets for *n* = 3

**Table S4** AGREEprep criteria, factors, weights and scores in the sustainability assessment of the optimised h-NADES UAE method (61 °C for 24 min, 1 extraction cycle)

| **Criteria** | Factor | Weight | Score |
| --- | --- | --- | --- |
| 1. Sample preparation placement | *ex situ* | 1 | 0.00 |
| 2. Hazardous materials | 0 mL | 5 | 1.00 |
| 3. Sustainability, renewability and reusability of materials | 50-75% sustainable and renewable reagents | 2 | 0.50 |
| 4. Waste | 0.1 g | 4 | 1.00 |
| 5. Size economy of the sample | 0.1 g | 2 | 1.00 |
| 6. Sample throughput | 35 Samples / hour | 3 | 0.84 |
| 7. Integration and automation | 3-stage, semi-automated | 2 | 0.38 |
| 8. Energy comsumption | 62 W·h / sample | 4 | 0.53 |
| 9. Post-sample preparation configuration for analysis | LC-MS | 1 | 0.25 |
| 10. Operator’s safety | 0 | 3 | 1.00 |
